# FonoTCS: validation of a tool for assessing clinical reasoning in Speech-Language pathology

**DOI:** 10.1590/2317-1782/e20240206en

**Published:** 2025-04-07

**Authors:** Ana Cristina Côrtes Gama, Roberto da Costa Quinino, Adriane Mesquita Medeiros, Patrícia Cotta Mancini, Aline Mansueto Mourão, Lara Gama Santos, Thais Helena Machado, Nayara Ribeiro Gomes

**Affiliations:** 1 Departamento de Fonoaudiologia, Faculdade de Medicina, Universidade Federal de Minas Gerais – UFMG - Belo Horizonte (MG), Brasil.; 2 Departamento de Estatística, Instituto de Ciências Exatas, Universidade Federal de Minas Gerais – UFMG - Belo Horizonte, MG, Brasil.; 3 Centro Federal de Educação Tecnológica de Minas Gerais – CEFET - Belo Horizonte (MG), Brasil.; 4 Programa de Pós-graduação (doutorado) em Ciências Fonoaudiológicas, Departamento de Fonoaudiologia, Faculdade de Medicina, Universidade Federal de Minas Gerais – UFMG - Belo Horizonte (MG), Brasil.

**Keywords:** Speech-Language Pathology, Clinical Reasoning, Clinical Diagnosis, Clinical Decision-Making, Students, Learning

## Abstract

**Purpose:**

To validate the internal structure of the Speech-Language Pathology Script Concordance Test (FonoTCS), which will be developed in a virtual, open-access format, to be used in the assessment of clinical reasoning among young professionals and students of speech-language pathology with a generalist background, speakers of Brazilian Portuguese.

**Methods:**

This is a study to validate the internal structure of the instrument. Twenty-five specialist speech-language pathologists, with more than 10 years of generalist clinical experience, and 35 students summoned for Enade participated. Both groups evaluated the 30 clinical cases with 120 items from FonoTCS. For the final selection of specialists who made up the sample, judges whose evaluations showed Z2 results >2 and Z<-2 distant from the modal response were removed. For the selection of items present in the final format of the test, those that remained had a Pearson correlation between the transformed scores of students for a given item and the sum of the transformed scores for all items, with a value greater than 0.05. The Cronbach's Alpha test was applied to measure the internal consistency of FonoTCS, and the score of each item was defined based on the aggregated score method.

**Results:**

The responses of 13 specialists were considered for the definition of the final test score. The final instrument had 88 items distributed across 28 clinical cases. The internal consistency was 0.903 with a 95% confidence interval expressed by 0.86|---|0.95. These values indicate a high internal consistency among the items of FonoTCS.

**Conclusion:**

FonoTCS is valid and reliable for use in evaluating the clinical reasoning of young professionals and speech-language pathology students with generalist training, who are Brazilian Portuguese speakers.

## INTRODUCTION

Clinical reasoning is the cognitive process in which a health professional uses the information contained in their knowledge and experience to guide their clinical practice^(1)^. It is a central theme in the diagnostic and therapeutic conduct of health professionals, and its concept has a complex and multidimensional nature^(2)^.

The use of qualified assessment tools in the curricula of undergraduate courses in the health area is essential to ensure that future professionals develop comprehensive and well-founded competencies. These tools can be of various formats, such as multiple-choice questions (MCQ), oral exams (OE), and matching questions (MQ), which primarily assess students’ cognitive mastery^(3)^. The structured objective clinical examination (SOCE), on the other hand, represents an approach that integrates the evaluation of both the cognitive process and the motor skills of students in simulated clinical contexts^(3)^.

These evaluative strategies, although valuable, still present limitations in their ability to reflect the complexity and dynamics of real clinical practice. The script concordance test (SCT) was developed in the late 90s, with the aim of evaluating the clinical reasoning of medical students in Canada^(4)^. It is a written simulation test in which various clinical scenarios are presented in a context of uncertainty, in routine clinical circumstances^(4)^.

The SCT is an assessment tool based on the theory of scripts derived from cognitive psychology and is based on the principle that, in order to assign meaning to a new situation, the information received activates a network of previously acquired knowledge and experiences - a script - which directs the selection, interpretation and memorization of this new information^(5)^.

In the clinical context, when evaluating a patient, a healthcare professional analyzes symptoms, signs, and details of the patient's environment^(4)^. These elements activate knowledge networks that contain information about these characteristics and their relationships with various diseases or clinical conditions (scripts) that direct to decision-making, either in the diagnostic or therapeutic process^(5)^.

The script is a derivation of a broader concept, that of schemes. Mental schemes are knowledge structures adapted to perform tasks efficiently^(5)^. Scripts are schemes associated with sequences of events that often occur in a specific order, and knowledge about different clinical conditions includes information about the spatio-temporal sequence of events in the development of the clinical picture^(4,5)^. By integrating this concept, the SCT assesses how professionals access these mental models or scripts to interpret and respond to complex clinical scenarios.

The question of how to evaluate the clinical reasoning of students in health-related courses during academic training arises continuously in curricula. Research^(3)^ comparing the performance of medical students with different assessment tools, concluded that students performed better on more familiar assessment tools such as MCQ, but most students considered the CP test better for evaluating learned content, and the SCT was best for testing clinical decision-making ability^(3)^.

Studies on the development of SCT in various areas of medicine^(6)^, nursing^(7)^, dentistry^(8)^, and physiotherapy^(9),^ have been widely disseminated, and the psychometric properties of validity and reliability of the tests have shown satisfactory performance.

In 2023, a SCT was developed for speech therapy students who speak Chilean Spanish^(10)^. The authors concluded that the test developed showed a low overall reliability. The stratified analysis by area of speech therapy (child and youth language; voice; orofacial motricity; language in adults and the elderly; audiology; and vestibular evaluation), and type of scenario (diagnosis, study, or intervention) offered a heterogeneous result, with variability in internal consistency values^(10)^.

This research relevance lies in the need to develop specific tools to evaluate clinical reasoning in speech therapy, where there is still a gap of validated instruments that can reflect the particularities of this professional practice.

The *Joint Committee on Standards for Educational and Psychological Testing* ^(11)^ proposes five sources of validity evidence: (1) content; (2) internal structure; (3) relationship to external measures; (4) pattern of response to items; and (5) consequential.

The FonoTCS is a SCT for the evaluation of clinical reasoning in speech therapy that proved to be a valid instrument from the point of view of content (clarity, relevance, and ethics)^(12)^.

This research aims to validate the internal structure of FonoTCS, to be used in the evaluation of clinical reasoning of young professionals and speech therapy students with general training, speakers of Brazilian Portuguese.

After the validation of its internal structure, FonoTCS will be made available in a virtual format and free access, and will advance to the external validation phase, including the practical application of the tool in an educational context, in order to evaluate its applicability in the training of speech therapy students with general training, speakers of Brazilian Portuguese.

## METHODS

This is a validation study of the internal structure of the instrument that follows the standards of the *Joint Committee on Standards for Educational and Psychological Testing* ^(11)^ and preparation of the SCT^(13-16)^. The research was approved by the Research Ethics Committee (COEP) of the Universidade Federal de Minas Gerais under Opinion number 5,824,852 and has already passed content validation^(12)^.

### Participants

Two distinct groups were invited to participate in the study for each validation stage. The first group was composed of specialists, consisting of 27 speech therapists with graduate degrees (*lato sensu or stricto sensu*) with more than 10 years of general clinical practice. The second group consisted of 52 speech therapy undergraduate students, summoned for the National Examination of Student Performance (Enade).

Both groups were informed about the objectives of the research and signed the Informed Consent Form (ICF). Subsequently, they were asked to answer the FonoTCS.

SCT questions avoid single “correct” or “consensus” answers. The score is based on an aggregate method that considers the variability of specialist responses to specific clinical situations^(13)^. The most common response among specialists (modal response) will be considered the “gold standard”, while other responses reflect different interpretations that may be clinically valuable, and these receive partial scores^(16)^.

Unlike conventional assessment tools, SCT recognizes that experienced clinicians can interpret data and respond to uncertain clinical situations in a variety of ways (within an acceptable range of clinical practice)^(15)^. Thus, to identify specialists and items of high disagreement in the FonoTCS, a posteriori analysis of the judges and items was conducted. This approach aims to define the final format of the FonoTCS and ensure the quality of the answers of the specialists and the items and is considered a practical and justifiable method to maintain the psychometric rigor of a test^(15)^.

The average estimated time to complete the FonoTCS was 60 minutes, and all responses were processed anonymously. All participants (specialists and students) were instructed to answer the FonoTCS in a single session, independently.

The initial format of FonoTCS, with validated content, was composed of 120 items distributed in 30 different clinical cases from six areas of speech therapy knowledge (audiology, language, orofacial motricity, dysphagia, voice, and collective health) with four items each^(12)^. Each of these clinical cases reflects distinct and complex situations, requiring the speech therapist to make specific and differentiated decisions, according to the particularities of each case.

Participants were instructed that each FonoTCS question comprises a scenario (description of the clinical case) with four questions (items) presented in three distinct parts^(14)^:

1. the first part (“if you're thinking about”) addresses a clinical decision of relevance;
2. the second part (“and you find”) introduces a clinical finding, such as sign, symptom, pre-existing condition, diagnostic image, or examination/test result;
3. the third part (“the hypothesis becomes”) consists of a five-point Likert scale that captures the decisions of the participants, in which 1 indicates a situation “practically ruled out/ totally contraindicated” and 5 a situation “practically certain/absolutely necessary”.

The participants' task was to determine the impact of the new discovery (second part) on the clinical decision (first part), in terms of direction (positive, negative, or neutral) and intensity (third part)^(16)^.

### Procedures

#### Specialists assessment

At this stage, of the 27 invited speech therapists, 25 (92.6%) evaluated the 120 items of the instrument. All received individual contact, with the explanations to answer the FonoTCS sent by *Google Forms*. The specialists had a period of 30 days to send the response.

#### Students assessment

Of the 52 students summoned to Enade, 35 (67.3%) answered FonoTCS. The participants answered the test in a university room, on individual computers. The test completion time for each student was recorded.

### Final format of FonoTCS

To define the final format of the test, an analysis was first performed to identify the judges who would be removed from the sample, from the calculation of the z-score^(17)^. Subsequently, the 120 items of the FonoTCS were evaluated, and those with a total item/item correlation lower than 0.05 by the Pearson correlation test were eliminated^(17)^.

For the data analysis, we used software available on the website of the University of Montreal for the preparation of SCT^(18)^.

#### Selection of specialist judges

The criterion used for the selection of speech therapists judges and definition of the “gold standard” response of the test was the standardized values Z_2_ and Z^(19)^.

Table 1 illustrates the initial evaluation process of the 25 specialists. The most appropriate response to each item was the modality of all specialists. Speech-language pathologists with a low percentage of responses equal to the item mode and/or if the sum of the distances of the responses in relation to the item mode was large were excluded from the study; i.e., the specialists whose evaluations presented results of Z_2_ >2 and Z<-2 away from the modal response.

**Table 1:** Evaluation of the specialists for each item of the FonoTCS (n=25)

**Panel**	**q1**	**q2**	**...**	**q118**	**q119**	**q120**
Specialist 1	1	2	...	1	2	2
Specialist 2	1	3	...	3	4	2
Specialist 3	2	1	...	1	5	4
Specialist 4	2	1	...	2	5	4
Specialist 5	4	2	...	1	5	1
Specialist 6	2	1	...	2	4	2
Specialist 7	2	1	...	1	2	1
Specialist 8	4	1	...	1	5	1
Specialist 9	1	1	...	5	2	1
Specialist 10	2	4	...	4	2	1
Specialist 11	5	4	...	4	5	4
Specialist 12	1	1	...	2	5	1
Specialist 13	3	2	...	3	5	1
Specialist 14	5	1	...	5	5	5
Specialist 15	1	3	...	3	5	1
Specialist 16	4	1	...	2	4	1
Specialist 17	2	1	...	2	5	1
Specialist 18	3	1	...	2	5	4
Specialist 19	2	5	...	1	5	2
Specialist 20	2	1	...	1	5	1
Specialist 21	1	1	...	1	5	1
Specialist 22	4	2	...	4	4	4
Specialist 23	2	1	...	3	5	2
Specialist 24	1	2	...	4	5	1
Specialist 25	4	1	...	5	5	4
modality	2	1	...	1	5	1

**Caption:** Q-item from FonoTCS

Table 2 presents the sum of the absolute distances of the responses in relation to the mode of each item (SD) and its respective standardized value (Z_2_), that is, the SD value subtracted from the SD column mean divided by the standard deviation of the SD column. It is also observed the percentage of responses equal to item mode (PAM) and its respective standardized value (Z).

**Table 2:** Sum of the distances (SD) and percentage of correct in relation to the mode (map) of the answers of the specialists for the items of the FonoTCS

	**SD**	**Z_2_**	**PAM**	**Z**
Specialist 1	83	-1.06	60.0%	0.950
Specialist 2	137	1.37	20.8%	-2.833
Specialist 3	92	-0.66	53.3%	0.306
Specialist 4	83	-1.06	62.5%	1.191
Specialist 5	124	0.79	41.7%	-0.821
Specialist 6	119	0.56	25.8%	-2.350
Specialist 7	100	-0.30	52.5%	0.225
Specialist 8	94	-0.57	55.0%	0.467
Specialist 9	138	1.42	45.8%	-0.419
Specialist 10	103	-0.16	57.5%	0.708
Specialist 11	151	2.00	42.5%	-0.741
Specialist 12	92	-0.66	56.7%	0.628
Specialist 13	113	0.29	42.5%	-0.741
Specialist 14	89	-0.79	62.5%	1.191
Specialist 15	79	-1.24	58.3%	0.789
Specialist 16	97	-0.43	54.2%	0.386
Specialist 17	110	0.16	50.8%	0.064
Specialist 18	90	-0.75	55.0%	0.467
Specialist 19	94	-0.57	50.8%	0.064
Specialist 20	99	-0.34	46.7%	-0.338
Specialist 21	104	-0.12	55.0%	0.467
Specialist 22	168	2.77	38.3%	-1.143
Specialist 23	92	-0.66	58.3%	0.789
Specialist 24	104	-0.12	54.2%	0.386
Specialist 25	109	0.11	53.3%	0.306

**Caption:** Z and Z_2_ - standardized values

Thus, measures of Z_2_ >2 and Z<-2 indicate assessments of specialists far from modality^(17)^. In this way, in the first round of interaction, four specialists (2, 6, 11 and 22) were withdrawn from the study. The entire analysis was redone without these specialists and, when necessary, new specialists were withdrawn. This process was terminated when no further measurements with Z values were detected_2_ >2 and Z<-2. In total, eight rounds of interaction were conducted that resulted in the elimination of 12 specialists from the study.

#### Items selection

The item was considered valid if the Pearson correlation between the transformed grades of the students for a given item, with the sum of the transformed grades for all items, was greater than 0.05^(20)^. The transformed grade of the students was equal to the frequency of the student's grade on the scale *Likert* in the specialist group, divided by the frequency of the specialist group modality.

Table 3 illustrates part of the students’ grades using the scale *Likert,* as well as the modality of the specialists and respective modality frequency.

**Table 3:** Students’ responses according to the scale *Likert* for each item of FonoTCS and modality value and modality frequency of the specialists

	**q1**	**q2**	**...**	**q119**	**q120**
Student 1	3	1	...	5	1
Student 2	2	3	...	5	4
Student 3	2	1	...	5	2
Student 4	3	2	...	5	1
Student 5	1	2	...	5	2
Student 6	3	2	...	5	1
...	...	...	...	...	...
Student 30	2	4	...	5	1
Student 31	2	4	...	5	1
Student 32	3	4	...	5	1
Student 33	2	2	...	4	3
Student 34	2	1	...	4	2
Student 35	1	1	...	2	2
modality Specialist	2	1	...	5	1
modality frequency	6	9	...	10	6
# Specialist 1 Note	3	9	...	0	6
# Specialist 2 Note	6	2	...	2	3
# Specialist 3 Note	1	0	...	0	0
# Specialist 4 Note	2	1	...	1	3
# Specialist 5 Note	1	1	...	10	1

**Caption:** Q-item from FonoTCS

Table 4 shows the transformed notes (T) and the respective sum of the transformed notes (SNT). For example, the grade 0.167 for Student 1 for item 1 is obtained by dividing the frequency of Grade 3 (from Table 3) in the specialist group by the frequency of the modality in the specialist group, that is, 1/6. The transformed grade 0.222 of Student 6 for Question 2 is obtained by fraction 2/9.

**Table 4:** Students’ transformed grades (NT) and sum of transformed grades (SNT) for each FonoTCS item

	**NTq1**	**NTq2**	**...**	**NTq119**	**NTq120**	**SNT**
Student 1	0.167	1.000	...	1.000	1.000	74.630
Student 2	1.000	0.000	...	1.000	0.500	67.995
Student 3	1.000	1.000	...	1.000	0.500	75.718
Student 4	0.167	0.222	...	1.000	1.000	63.192
Student 5	0.500	0.222	...	1.000	0.500	86.263
Student 6	0.167	0.222	...	1.000	1.000	84.956
...	...	...	...	...	...	...
Student 30	1.000	0.111	...	1.000	1.000	90.274
Student 31	1.000	0.111	...	1.000	1.000	80.157
Student 32	0.167	0.111	...	1.000	1.000	90.977
Student 33	1.000	0.222	...	0.100	0.000	65.506
Student 34	1.000	1.000	...	0.100	0.500	90.085
Student 35	0.500	1.000	...	0.200	0.500	74.453

**Caption:** Q-item from FonoTCS

After transforming the students’ grades, all 120 correlations between the transformed grades recorded in the columns of Table 4 (for example, NTq1) with the SNT column were calculated. Pearson correlations greater than 0.05 indicate that the item should remain in the study^(20)^.

At this stage, of the 120 items, 27 items were removed from the study because they presented correlations lower than 0.05. The entire process was repeated until all correlations were greater than 0.05. Another five items were removed due to the recommendation that each scenario (clinical case) needs to have at least two items that characterize it^(14)^ therefore, after four rounds of interactions, 88 items composed the final instrument.

### Data analysis

After the withdrawal of specialists and items and definition of the final format of the FonoTCS, the Cronbach's Alpha test was applied to measure the internal consistency of the test.

FonoTCS responses were defined using the aggregate score method^(15)^. The score was based on the distribution of responses among specialists for each item. Total credits were given to the modal response (more chosen by the specialists), and partial credits were assigned to other responses, according to the proportion of choice of speech therapists specialists. Answers not chosen by the judges received zero scores.

Subsequently, the results of the students 'and specialists' responses were compared to evaluating the responses of both groups, based on descriptive analysis of the data.

Upon completion of the FonoTCS, the respondent will receive a final grade. The final total score for the test is the sum of the points obtained on each item. This value is divided by the number of test items and at the end multiplied by 100 to obtain a percentage score^(5)^.

## RESULTS

After defining the final format of the FonoTCS, 13 specialists (52%) of the 25 speech therapists who answered the FonoTCS participated in the study, being 12 women (92.0%) and one man (8.0%) with a mean age of 41.7 years (SD=6.43). Regarding the level of education, the sample had one specialist (08%), eight Masters (61%) and four doctors (31%), with an average time of general clinical practice of 17.9 years (SD=6.3).

Of the 35 students of the speech therapy course, students of the 9th and 10th periods summoned to Enade, most were female (n=32; 91.4%) with an average age of 25.5 years (SD=7.76). The Mean Time for students to perform the FonoTCS was 58.9 minutes (SD = 8.93).

The final instrument presented 88 items distributed in 28 clinical cases, six cases for the areas of knowledge of speech therapy of audiology, language, orofacial motricity / dysphagia, and five cases for the areas of voice and public health.

**Figure 1:**
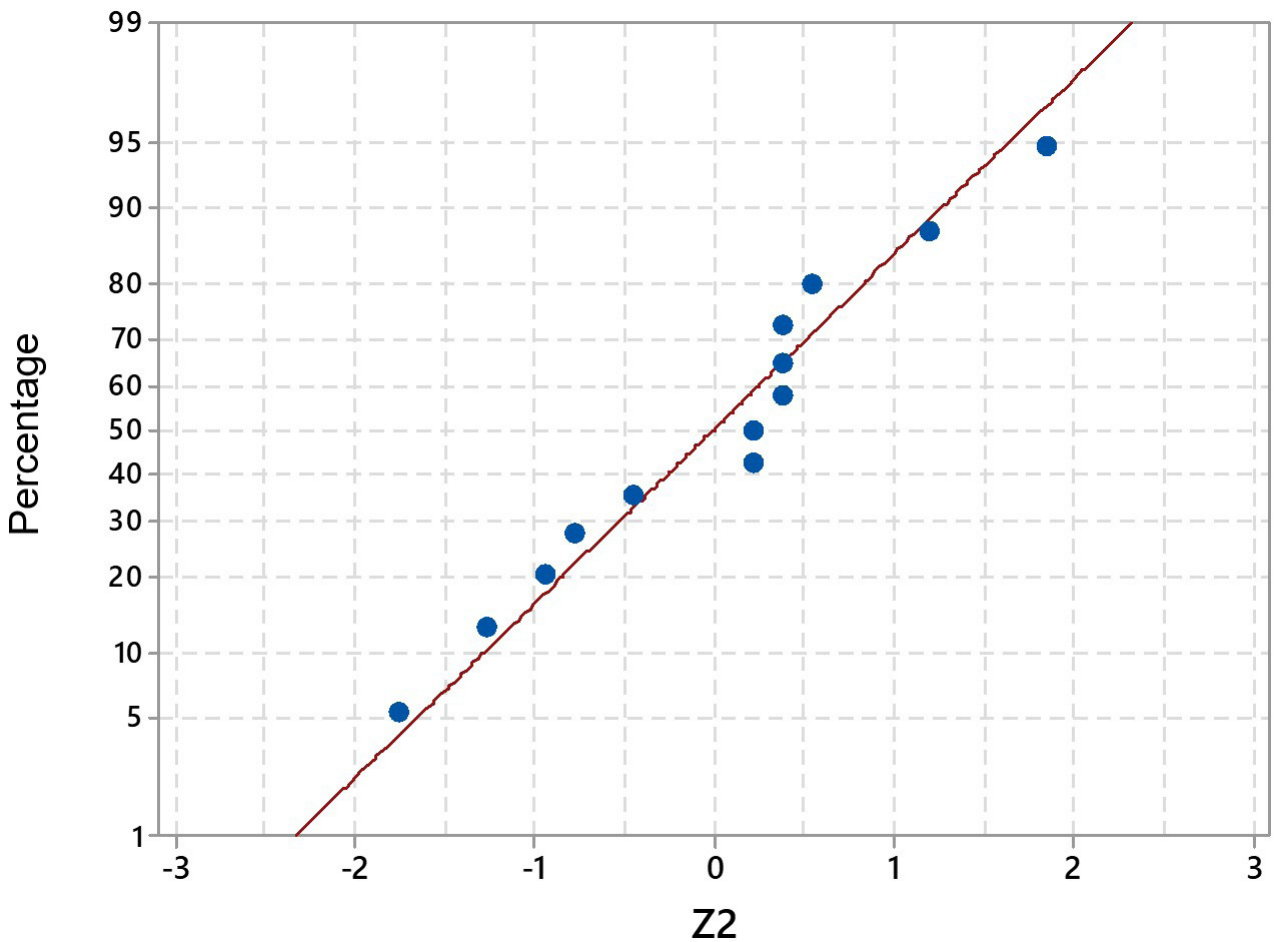
QQ Plot graph for standardized z data_2_ of the specialists who remained in the final version (n=13)

**Figure 2:**
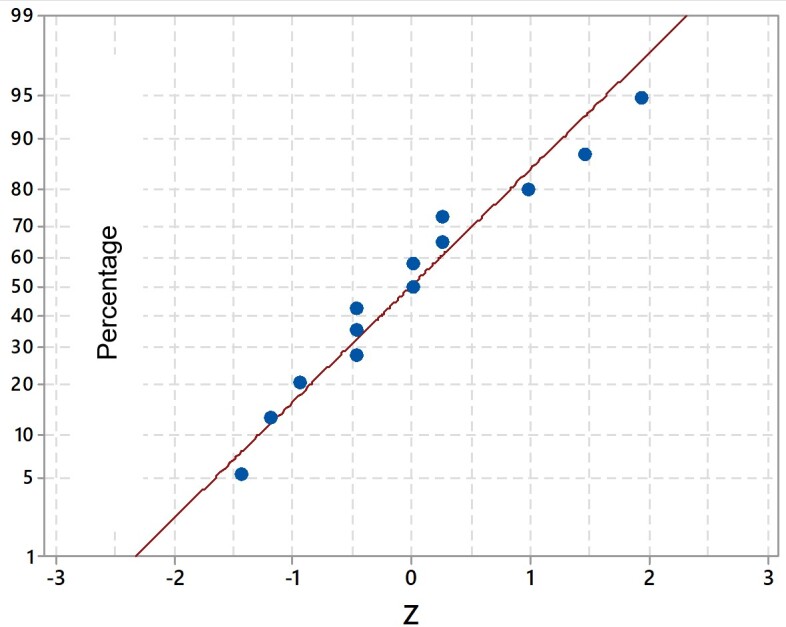
QQ chart *Plot* for standardized z data of the specialists who remained in the final version (n=13)

Figures 1 and 2 illustrate the normal probability plots for the Z values_2_ and Z of the 13 specialists, respectively. It was observed that there is no evidence to reject that the data are normal at the significance level 5% (p-value 0.05 for the Anderson Darling test) and no specialist presented a value of Z_2_ >2 and / or Z< -2.

Using the transformed data described in Table 4 with the 88 items that remained in the study, a Cronbach's Alpha equal to 0.903 was obtained with a 95% confidence interval expressed by 0,86|---|0,95. These values indicate a high internal consistency between the items of the FonoTCS^(21)^.

**Figure 3:**
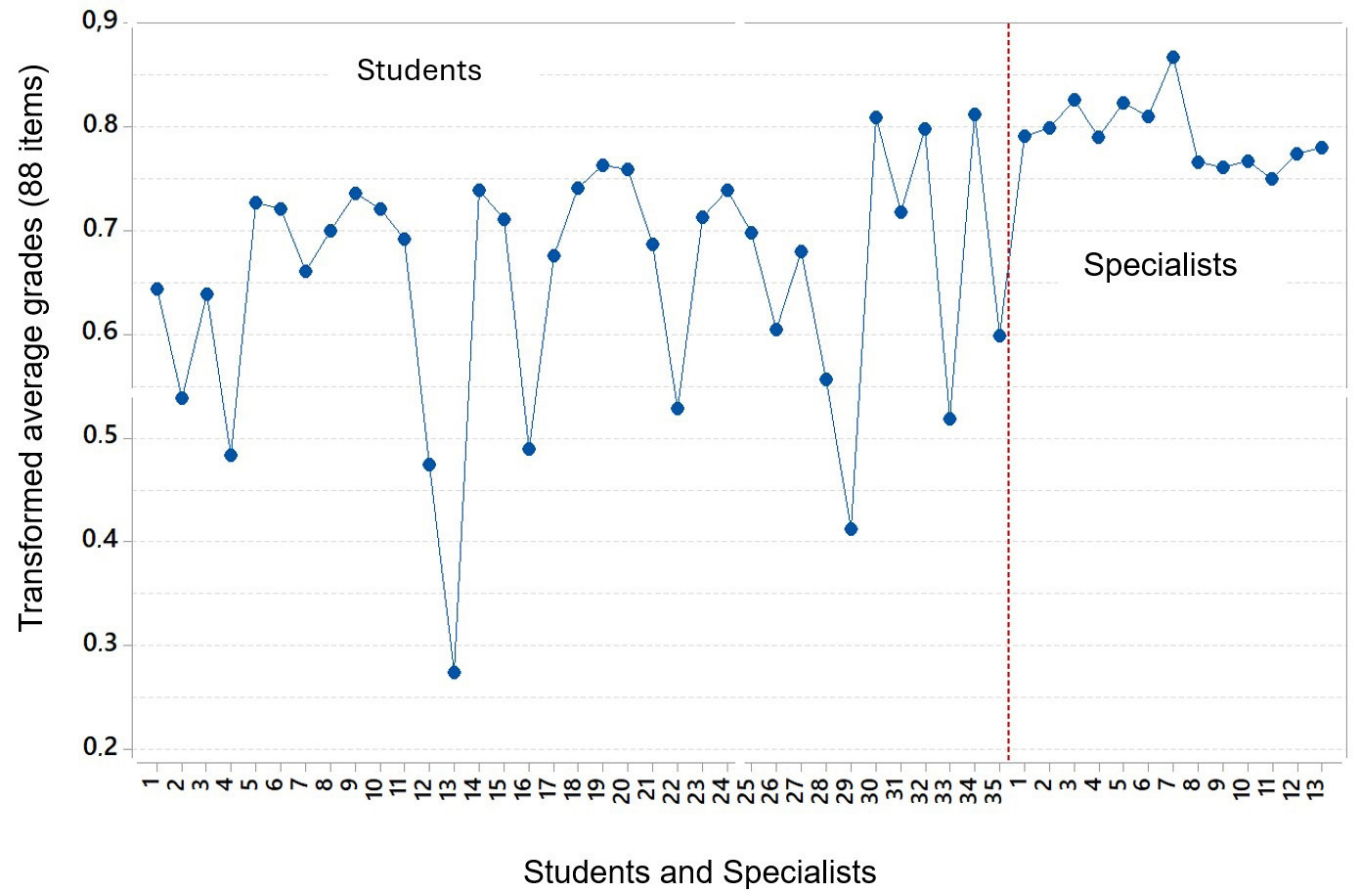
Assessment of the performance of students and specialists

We can observe in Figure 3 that the 13 specialists presented, as expected, a higher average performance when compared to the students (n=35) in addition to a lower variability. In relation to students, there was great variability and an average performance lower than that of specialists.

We can observe that certain students (19, 20, 31, 32 and 34) presented a very good performance comparable to that of specialists and can be called “specialist students” considering the evaluation of the 88 items and corroborating an adequate learning process. On the other hand, some students (2, 4, 12, 13, 16, 22, 29 and 33) presented an average performance, in descriptive terms, lower than that of the specialists.

The final format of FonoTCS is available for free access on the page of the Faculty of Medicine of the Federal University of Minas Gerais, by link https://fonotcs.medicina.ufmg.br/.

## DISCUSSION

The results of this research indicated that it is possible to evaluate the clinical reasoning of students and young speech therapists with generalist training. The FonoTCS was considered appropriate to evaluate the ability of the examinees to generate hypotheses and decide on decision-making in the Diagnostic and therapeutic process, in contexts of uncertainty in the speech therapy field.

The initial format of FonoTCS, with validated content^(12)^, was composed of 120 items distributed in 30 clinical cases from six areas of knowledge of speech therapy (audiology, language, orofacial motor skills, dysphagia, voice, and public health) with four items each. Its final format consisted of 28 clinical cases with 88 items, six cases for the areas of knowledge of audiology, language, orofacial motricity / dysphagia and five cases for the areas of voice and public health.

The final configuration of FonoTCS was defined in two stages of analysis: 1) selection of specialists from the standardized values Z_2_ and Z; and 2) removal of items with a total item/item correlation of less than 0.05. A unique feature of Script Concordance tests (SCTs) is the formation of a panel of reference specialists, which has the function of determining the final score of the test^(15,17)^.

The guidelines for the elaboration of the SCTs indicate that, to obtain reliable tests, 10 to 15 judges are needed to achieve adequate estimates of reliability, with a marginal benefit when having more than 20 specialists^(13-17,22)^. Of the 25 speech therapists who responded to the FonoTCS, 13 specialists were kept in the final analysis for the definition of the reference responses of the test, as suggested by the literature^(22)^.

After the analysis of the item/total item correlation, 27 items (22.5%) were removed, and then five were left with scenarios (clinical cases) containing only two items, totaling 32 (26.7%) items removed. According to literature^(14)^, it is estimated that 25% of the items in the elaboration of the SCTs were removed. The use of three to four items per case in the SCT is justified by theoretical and psychometric issues of the test^(23)^. SCT guidelines recommend 20 to 25 cases with at least 75 items^(13-16)^, to achieve acceptable reliability^(14)^. The FonoTCS respected all the recommendations^(13-16)^ to prepare the SCTs, presenting a format composed of 28 clinical cases with 88 items, with three to four items per case.

The test response time is approximately 60 Minutes. Research^(22)^ states that SCTs with test times between 60 and 90 minutes have been shown to produce adequate scoring reliability^(22)^.

Cronbach’s Alpha obtained was 0.903, with a 95% confidence interval ranging between 0.86 and 0.95. These values indicate a high internal consistency between FonoTCS items^(21)^. Considering that the 88 items of the FonoTCS are distributed in 28 clinical cases, each representing distinct and complex situations in different areas of speech therapy, this diversity ensures that the high internal consistency is not the result of homogeneous items, minimizing the risk of artificial inflation of Cronbach's Alpha, and reinforcing the validity of the instrument to evaluate the clinical reasoning of speech therapists with generalist training. Another aspect to highlight is that FonoTCS was submitted to content validation and proved to be a valid instrument in terms of clarity, relevance, and ethical aspects^(12)^.

Different validated SCTs to evaluate clinical reasoning in medicine, covering clinical situations of Rheumatology^(24)^, Otolaryngology^(25)^ and anesthesiology^(26)^, had Cronbach's alpha values of 0.82^(24,25)^ and 0.79^(26)^, all within the range of 0.75 to 0.80 recommended by the literature^(14)^.

In speech therapy, a SCT was developed for Chilean Spanish-speaking students^(10)^ with 80 items and a reliability of 0.67 (minimum = 0.34; maximum = 0.84). The authors conclude that, although the test presented a low overall reliability, the stratification by area of specialty of each scenario offered a heterogeneous panorama^(10)^. It is noteworthy that the authors^(10)^ have not followed all the rules for the preparation of the SCT^(13-16)^, which may have influenced the results found.

The internal consistency results of the FonoTCS of 0.903 demonstrate that the test items are correlated with each other and, therefore, coherently measure the same construct, defining the FonoTCS as an evaluation instrument with reliability in the scores obtained^(21)^, and results within the expected for the SCTs^(14,15)^.

The answers provided by the specialist speech therapists defined the final score of the FonoTCS based on the aggregate score method^(13-16)^. To exemplify this method, suppose a 15-member panel was asked to answer the first question of the test, and eight members selected the answer +1, five members selected the answer +2, and two members selected the answer 0. The score for this item would be answer +1, 1 point (8/8), because it is the modal answer; answer +2, 0.625 points (5/8); answer 0, 0.25 points (2/8); and answers -1 and -2 with 0 points.

Variability in the scoring of panel responses is an inherent characteristic of SCT and has been shown to be a key element in its discriminative capacity and, consequently, in its validity^(16)^.

The aggregate score method described above is the most used method in SSC^(27)^. However, it is important to recognize that the optimal scoring method for SCT is still debated^(28)^. The aggregate score method also requires psychometric research on aspects such as consensus level, scoring scale and its relationship with the discrimination between the responses of experienced professionals and young clinicians^(13)^.

Despite the debate about the best analysis method to be used in SCTs^(28)^, variability in response scores is essential for these tests. The theoretical foundation of SCTs is based on the development of scripts from the individual experience of the clinician with patients, which results in a significant degree of idiosyncrasy, especially in less common cases. Therefore, the variability in responses, based on the aggregate score method score, is central to the argument about the validity of SSC in the ability to measure clinical reasoning^(15)^.

As described, the aggregate score method weights responses based on their frequency in the panel of specialists, allowing a score that reflects the collective opinion, with proportional weights assigned to less frequent responses.

One of the main positive impacts of this method is its ability to capture variability in specialists’ responses, which contributes to fine discrimination between distinct levels of clinical reasoning. This aspect is fundamental for the validity of the FonoTCS, since the observed variability reflects the different experiences and training of professionals, especially in more rare or complex clinical cases^(16)^. This discriminative capacity reinforces the argument that FonoTCS is sensitive to the nuances of clinical reasoning, a central characteristic of SSC^(13-16)^.

On the other hand, a potentially negative impact of the aggregate score method is that it can introduce subjectivity into the scores, especially when there is a high degree of dispersion in the specialists’ responses. This variation can make it difficult to create a clear standard for all cases, which raises the need for more psychometric research, such as investigating the level of consensus necessary to define correct answers and analyzing the relationship between this consensus and discrimination between experienced clinicians and young professionals^(13)^.

Therefore, while the aggregate score method offers a robust way to reflect the diversity of responses and clinical experiences in FonoTCS, we recognize that it also presents challenges that require additional investigation. However, the results of this study indicate that this approach is effective in capturing the complexity of speech therapy clinical reasoning, contributing to the validity of the instrument^(17,28)^.

FonoTCS will be presented in electronic format, with free access, to present exams by image and to return the results to the examinees in real time^(14,15)^. At the end of the FonoTCS, the respondent will receive a final grade calculated as follows: the total score is obtained by adding the points of each item of the test. This sum is then divided by the total number of test items and multiplied by 100 to obtain a percentage score and facilitate the interpretation of the result^(5)^.

When comparing the results of the 13 specialist speech therapists with those of the 35 students, the specialists demonstrated a higher performance with a significantly lower variability. Some students exhibited excellent performance, comparable to that of specialists, while others showed descriptively lower performance than specialists. Such results are expected and demonstrate the ability of SSCs to discriminate distinct levels of clinical reasoning^(13-16)^.

As a limitation of this study, it is important to highlight that the elaboration of a SCT requires a significant investment of human resources, time, and dedication. In a context where efficiency is crucial, rapid, and effective adaptations of assessment methods are essential to ensure the quality of teaching of health professionals. A recent study^(29)^ explored the development of a SCT supported by artificial intelligence (AI), pointing out that ChatGPT can be a promising tool in creating these tests. This research^(29)^ opens new prospects for further research in the field of SCT.

The current scenario highlights the need to develop new SCTs to evaluate clinical reasoning in the various specialties of speech therapy. In the context of generalist training, FonoTCS proved to be a useful and innovative tool for evaluating the clinical reasoning of Brazilian Portuguese-speaking speech therapists.

FonoTCS proved to be effective as an evaluative tool, but it also plays a significant role in the teaching-learning process. By exposing students to real and challenging clinical scenarios, it encourages the practical application of theoretical knowledge, promoting the development of more robust and integrated clinical reasoning.

This active and contextual approach allows students to reflect on their decisions and improve their decision-making skills, essential aspects for their professional training. The relevance of the FonoTCS in the educational process lies in its ability to simulate situations of uncertainty that are frequent in clinical practice, contributing to more complete training and aligned with the demands of the labor market and the reality of speech therapy.

## CONCLUSION

The FonoTCS is valid and reliable to be used in the evaluation of clinical reasoning of young professionals and speech therapy students with generalist training, speakers of Brazilian Portuguese.
