# Supplementary material for: FonoTCS: validation of a tool for assessing clinical reasoning in Speech-Language pathology
Source: Codas. 2025 Apr 7;37(3):e20240206. doi: 10.1590/2317-1782/e20240206en (PMC11991717; doi:10.1590/2317-1782/e20240206en)
Supplement: Translation

# Translation: FonoTCS: validação de uma ferramenta para avaliação do raciocínio clínico em Fonoaudiologia

**DOI:** 10.1590/2317-1782/e20240206pt

**Authors:** Ana Cristina Côrtes Gama, Roberto da Costa Quinino, Adriane Mesquita Medeiros, Patrícia Cotta Mancini, Aline Mansueto Mourão, Lara Gama Santos, Thais Helena Machado, Nayara Ribeiro Gomes

**Affiliations:** 1 Departamento de Fonoaudiologia, Faculdade de Medicina, Universidade Federal de Minas Gerais – UFMG - Belo Horizonte (MG), Brasil.; 2 Departamento de Estatística, Instituto de Ciências Exatas, Universidade Federal de Minas Gerais – UFMG - Belo Horizonte, MG, Brasil.; 3 Centro Federal de Educação Tecnológica de Minas Gerais – CEFET - Belo Horizonte (MG), Brasil.; 4 Programa de Pós-graduação (doutorado) em Ciências Fonoaudiológicas, Departamento de Fonoaudiologia, Faculdade de Medicina, Universidade Federal de Minas Gerais – UFMG - Belo Horizonte (MG), Brasil.

**Keywords:** Fonoaudiologia, Raciocínio Clínico, Diagnóstico Clínico, Tomada de Decisão Clínica, Estudantes, Aprendizagem

## Abstract

**Objetivo:**

Validar a estrutura interna do Teste de Concordância de Scripts em Fonoaudiologia (FonoTCS) que será desenvolvido em formato virtual com acesso livre, para ser utilizado na avaliação do raciocínio clínico de jovens profissionais e estudantes de fonoaudiologia com formação generalista, falantes do português brasileiro.

**Método:**

Trata-se de estudo de validação de estrutura interna de instrumento. Participaram 25 fonoaudiólogos especialistas, com mais de 10 anos de experiência clínica generalista e 35 estudantes convocados para o Enade. Ambos os grupos avaliaram os 30 casos clínicos com 120 itens do FonoTCS. Para a seleção final dos especialistas que compuseram a amostra, foram retirados os juízes cujas avaliações apresentavam resultados de Z2 >2 e Z<-2 distantes da resposta modal. Para a seleção dos itens presentes no formato final do teste, permaneceram aqueles que, na correlação de Pearson entre as notas transformadas dos estudantes para um determinado Item, com a soma das notas transformadas para todos os Itens, obtiveram valor superior a 0,05. O teste Alfa de Cronbach foi aplicado para medir a consistência interna do FonoTCS e a pontuação de cada item foi definida a partir do método de escore agregado.

**Resultados:**

As respostas de 13 fonoaudiólogos foram consideradas para definição da pontuação final do teste. O instrumento final apresentou 88 itens distribuídos em 28 casos clínicos. A consistência interna foi igual a 0,903 com intervalo de confiança de 95% expresso por 0,86|---|0,95. Estes valores indicam uma alta consistência interna entre os itens do FonoTCS.

**Conclusão:**

O FonoTCS é válido e confiável para ser utilizado na avaliação do raciocínio clínico de jovens profissionais e estudantes de fonoaudiologia com formação generalista, falantes do português brasileiro.

## INTRODUÇÃO

O raciocínio clínico é conceituado como o processo cognitivo no qual um profissional de saúde utiliza as informações contidas em seu conhecimento e experiência para guiar sua prática clínica^(1)^. É um tema central na conduta diagnóstica e terapêutica dos profissionais de saúde, e seu conceito possui uma natureza complexa e multidimensional^(2)^.

O uso de ferramentas de avaliação qualificada nos currículos dos cursos de graduação na área da saúde é fundamental para assegurar que os futuros profissionais desenvolvam competências abrangentes e bem fundamentadas. Estas ferramentas podem assumir diversos formatos, como perguntas de múltipla escolha (PME), exames orais (EO) e perguntas de correspondência (PC), que avaliam principalmente o domínio cognitivo dos alunos^(3)^. Já o Exame Clínico Objetivo Estruturado (OSCE) representa uma abordagem que integra a avaliação, tanto do processo cognitivo quanto das habilidades motoras dos estudantes, em contextos clínicos simulados^(3)^.

Estas estratégias avaliativas, embora valiosas, ainda apresentam limitações em sua capacidade de refletir a complexidade e a dinâmica da prática clínica real. O Teste de concordância de *script* (TCS) foi desenvolvido no final da década de 90, com o objetivo de avaliar o raciocínio clínico de estudantes de medicina no Canadá^(4)^. É um teste de simulação escrita em que vários cenários clínicos são apresentados em um contexto de incerteza, em circunstâncias clínicas rotineiras^(4)^.

O TCS é uma ferramenta de avaliação baseada na teoria de *scripts* derivada da psicologia cognitiva e baseia-se no princípio de que, para atribuir significado a uma nova situação, as informações recebidas ativam uma rede de conhecimentos e de experiências previamente adquiridos - um *script* - que direciona a seleção, interpretação e memorização dessas novas informações^(5)^.

No contexto clínico, ao avaliar um paciente, um profissional de saúde analisa sintomas, sinais e detalhes do ambiente do paciente^(4)^. Esses elementos ativam redes de conhecimento que contêm informações sobre essas características e suas relações com diversas doenças ou condições clínicas (*scripts*) que direcionam para a tomada de decisão, seja no processo diagnóstico ou terapêutico^(5)^.

O conceito de *script* é uma derivação de um conceito mais amplo, o de esquemas. Esquemas mentais são estruturas de conhecimento adaptadas para realizar tarefas de maneira eficiente^(5)^. *Scripts* são esquemas associados a sequências de eventos que ocorrem frequentemente em uma ordem específica, e o conhecimento sobre diferentes condições clínicas inclui informações sobre a sequência espaço-temporal de eventos no desenvolvimento do quadro clínico^(4,5)^. Ao integrar esse conceito, o TCS avalia como os profissionais acessam esses modelos mentais ou *scripts* para interpretar e responder a cenários clínicos complexos.

A questão de como avaliar o raciocínio clínico de estudantes da área da saúde durante a formação acadêmica surge continuamente nos currículos. Pesquisa^(3)^ que comparou a performance de estudantes de medicina com diferentes ferramentas avaliativas, concluiu que os estudantes tiveram um desempenho melhor em ferramentas de avaliação mais familiar como PME, mas a maioria dos estudantes consideraram o teste de PC melhor para avaliar o conteúdo aprendido, e o TCS testou melhor a habilidade clínica de tomada de decisão^(3)^.

Estudos sobre o desenvolvimento do TCS em diversas áreas da medicina^(6)^, enfermagem^(7)^, odontologia^(8)^, e fisioterapia^(9),^ têm sido amplamente divulgados e as propriedades psicométricas de validade e confiabilidade dos testes têm mostrado um bom desempenho.

Em 2023 foi desenvolvido um TCS para estudantes de fonoaudiologia falantes do espanhol chileno^(10)^. Os autores concluíram que o teste desenvolvido apresentou uma baixa confiabilidade geral. A análise estratificada por área de atuação fonoaudiológica (linguagem infanto-juvenil; voz; motricidade orofacial; linguagem em adultos e idosos; audiologia; e avaliação vestibular), e tipo de cenário (diagnóstico, estudo ou intervenção) ofereceu um resultado heterogêneo, com variabilidade nos valores de consistência interna^(10)^.

A relevância desta pesquisa reside na necessidade de desenvolver ferramentas específicas para avaliar o raciocínio clínico na Fonoaudiologia, onde ainda há uma lacuna de instrumentos validados que sejam capazes de refletir as particularidades dessa prática profissional.

O *Joint Committee on Standards for Educational and Psychological Testing* ^(11)^ propõe cinco fontes de evidência de validade: (1) conteúdo; (2) estrutura interna; (3) relação com medidas externas; (4) padrão de resposta aos itens; e (5) consequencial.

O FonoTCS é um TCS para a avaliação do raciocínio clínico em fonoaudiologia, que mostrou ser um instrumento válido do ponto de vista do conteúdo (clareza, pertinência e ética)^(12)^.

Esta pesquisa objetiva validar a estrutura interna do FonoTCS, para ser utilizado na avaliação do raciocínio clínico de jovens profissionais e estudantes de fonoaudiologia com formação generalista, falantes do português brasileiro.

Após a validação de sua estrutura interna, o FonoTCS será disponibilizado em formato virtual de acesso livre e avançará para a fase de validação externa, incluindo a aplicação prática da ferramenta em contexto educacional, visando avaliar sua aplicabilidade na formação de estudantes de fonoaudiologia com formação generalista, falantes do português brasileiro.

## MÉTODO

Trata-se de estudo de validação de estrutura interna de instrumento que segue as normas do *Joint Committee on Standards for Educational and Psychological Testing* ^(11)^ e de elaboração do TCS^(13-16)^. A pesquisa foi aprovada pelo Comitê de Ética em Pesquisa (COEP) da Universidade Federal de Minas Gerais sob parecer número 5.824.852 e já passou pela validação de conteúdo^(12)^.

### Participantes

Foram convidados a participar do estudo dois grupos distintos para cada etapa da validação. O primeiro grupo foi composto por especialistas, constituído por 27 fonoaudiólogos com pós-graduação (*lato sensu ou stricto sensu*) e mais de 10 anos de atuação clínica generalista. O segundo grupo, foi constituído por 52 estudantes do curso de graduação em Fonoaudiologia de uma universidade, convocados para o Exame Nacional de Desempenho de Estudantes (Enade).

Ambos os grupos foram esclarecidos com relação aos objetivos da pesquisa e assinaram o Termo de Consentimento Livre e Esclarecido (TCLE). Posteriormente, foram solicitados a responderem o FonoTCS.

As perguntas do TCS evitam respostas únicas “corretas” ou de “consenso”. A pontuação é baseada em um método agregado que considera a variabilidade das respostas de especialistas a situações clínicas específicas^(13)^. A resposta mais comum entre os especialistas (resposta modal) será considerada o “padrão ouro”, enquanto outras respostas refletem interpretações diferentes que podem ser clinicamente valiosas e estas recebem pontuações parciais^(16)^.

Diferente das ferramentas de avaliação convencionais, o TCS reconhece que clínicos experientes podem interpretar dados e responder a situações clínicas incertas de maneiras variadas (dentro de um intervalo aceitável de prática clínica)^(15)^. Desta forma, para identificar especialistas e itens de alta discordância no FonoTCS, realizou-se uma análise a posteriori dos juízes e dos itens. Essa abordagem objetivou definir o formato final do FonoTCS, e garantir a qualidade das respostas dos especialistas e dos itens, e é considerada um método prático e justificável para manter o rigor psicométrico de um teste^(15)^.

O tempo médio estimado para o preenchimento do FonoTCS foi de 60 minutos e todas as respostas foram processadas anonimamente. Todos os participantes (especialistas e estudantes) foram orientados a responder o FonoTCS em uma única sessão, de forma independente.

O formato inicial do FonoTCS, com conteúdo validado, foi composto por 120 itens distribuídos em 30 casos clínicos distintos de seis áreas de conhecimento da Fonoaudiologia (audiologia, linguagem, motricidade orofacial, disfagia, voz e saúde coletiva) com quatro itens cada^(12)^. Cada um desses casos clínicos reflete situações distintas e complexas, exigindo que o fonoaudiólogo tome decisões específicas e diferenciadas, de acordo com as particularidades de cada caso.

Os participantes receberam a orientação de que cada questão do FonoTCS compreende um cenário (descrição do caso clínico) com quatro perguntas (itens) apresentadas em três partes distintas^(14)^:

1. A primeira parte (“se você está pensando em”) aborda uma decisão clínica de relevância;
2. A segunda parte (“e você encontra”) introduz um achado clínico, como um sinal, sintoma, condição pré-existente, imagem diagnóstica ou resultado de exame/teste;
3. A terceira parte (“a hipótese se torna”) consiste em uma escala Likert de cinco pontos que captura as decisões dos participantes, no qual 1 indica situação “praticamente descartada/ totalmente contraindicada” e 5 uma situação “praticamente certa/absolutamente necessária”.

A tarefa dos participantes foi determinar o impacto da nova descoberta (segunda parte) na decisão clínica (primeira parte), em termos de direção (positiva, negativa ou neutra) e intensidade (terceira parte)^(16)^.

### Procedimentos

#### Avaliação dos especialistas

Nesta etapa, dos 27 fonoaudiólogos convidados, 25 (92,6%) avaliaram os 120 itens do instrumento. Todos receberam contato individual, com as explicações para responder o FonoTCS enviado pelo *GoogleForms*. Os especialistas tiveram um prazo de 30 dias para envio da resposta.

#### Avaliação dos estudantes

Dos 52 estudantes convocados para o Enade, 35 (67,3%) responderam o FonoTCS. Os participantes responderam o teste em uma sala da Universidade, em computadores individuais. O tempo de realização do teste para cada estudante foi mensurado.

### Formato final do FonoTCS

Para definição do formato final do teste, primeiramente se realizou uma análise para identificar os juízes que seriam retirados da amostra, a partir do cálculo do z-score^(17)^. Posteriormente foram avaliados os 120 itens do FonoTCS, e aqueles com correlação item/item total menor que 0,05 pelo teste de correlação de Pearson, foram eliminados^(17)^.

Para a análise dos dados se utilizou um *software* disponível no site da Universidade de Montreal para a elaboração de TCS^(18)^.

#### Escolha dos juízes especialistas

O critério utilizado para a seleção dos fonoaudiólogos juízes e definição da resposta “padrão ouro” do teste foi os valores padronizados Z_2_ e Z^(19)^.

A Tabela 1 ilustra o processo inicial de avaliação dos 25 especialistas. A resposta mais adequada para cada item foi a moda de todos os especialistas. Foram excluídos do estudo aqueles fonoaudiólogos com percentual baixo de respostas iguais a moda do item e/ou se a soma das distâncias das respostas em relação à moda do item foi grande, ou seja, foram retirados os especialistas cujas avaliações apresentavam resultados de Z_2_ >2 e Z<-2 distantes da resposta modal.

**Tabela 1:** Avaliação dos especialistas para cada item do FonoTCS (n=25)

**Painel**	**q1**	**q2**	**...**	**q118**	**q119**	**q120**
Especialista 1	1	2	...	1	2	2
Especialista 2	1	3	...	3	4	2
Especialista 3	2	1	...	1	5	4
Especialista 4	2	1	...	2	5	4
Especialista 5	4	2	...	1	5	1
Especialista 6	2	1	...	2	4	2
Especialista 7	2	1	...	1	2	1
Especialista 8	4	1	...	1	5	1
Especialista 9	1	1	...	5	2	1
Especialista 10	2	4	...	4	2	1
Especialista 11	5	4	...	4	5	4
Especialista 12	1	1	...	2	5	1
Especialista 13	3	2	...	3	5	1
Especialista 14	5	1	...	5	5	5
Especialista 15	1	3	...	3	5	1
Especialista 16	4	1	...	2	4	1
Especialista 17	2	1	...	2	5	1
Especialista 18	3	1	...	2	5	4
Especialista 19	2	5	...	1	5	2
Especialista 20	2	1	...	1	5	1
Especialista 21	1	1	...	1	5	1
Especialista 22	4	2	...	4	4	4
Especialista 23	2	1	...	3	5	2
Especialista 24	1	2	...	4	5	1
Especialista 25	4	1	...	5	5	4
Moda	2	1	...	1	5	1

**Legenda:** q – item do FonoTCS

A Tabela 2 apresenta a soma das distâncias absolutas das respostas em relação a moda de cada item (SD) e seu respectivo valor padronizado (Z_2_), ou seja, o valor de SD subtraído da média da coluna de SD dividido pelo desvio padrão da coluna de SD. Observa-se ainda, o percentual de respostas iguais a moda do item (PAM) e seu respectivo valor padronizado (Z).

**Tabela 2:** Soma das distâncias (SD) e percentual de acerto em relação a moda (PAM) das respostas dos especialistas para os itens do FonoTCS

	**SD**	**Z_2_**	**PAM**	**Z**
Especialista 1	83	-1,06	60,0%	0,950
Especialista 2	137	1,37	20,8%	-2,833
Especialista 3	92	-0,66	53,3%	0,306
Especialista 4	83	-1,06	62,5%	1,191
Especialista 5	124	0,79	41,7%	-0,821
Especialista 6	119	0,56	25,8%	-2,350
Especialista 7	100	-0,30	52,5%	0,225
Especialista 8	94	-0,57	55,0%	0,467
Especialista 9	138	1,42	45,8%	-0,419
Especialista 10	103	-0,16	57,5%	0,708
Especialista 11	151	2,00	42,5%	-0,741
Especialista 12	92	-0,66	56,7%	0,628
Especialista 13	113	0,29	42,5%	-0,741
Especialista 14	89	-0,79	62,5%	1,191
Especialista 15	79	-1,24	58,3%	0,789
Especialista 16	97	-0,43	54,2%	0,386
Especialista 17	110	0,16	50,8%	0,064
Especialista 18	90	-0,75	55,0%	0,467
Especialista 19	94	-0,57	50,8%	0,064
Especialista 20	99	-0,34	46,7%	-0,338
Especialista 21	104	-0,12	55,0%	0,467
Especialista 22	168	2,77	38,3%	-1,143
Especialista 23	92	-0,66	58,3%	0,789
Especialista 24	104	-0,12	54,2%	0,386
Especialista 25	109	0,11	53,3%	0,306

**Legenda:** Z e Z_2_ - valores padronizados

Assim, medidas de Z_2_ >2 e Z<-2 indicam avaliações de especialistas distantes da moda^(17)^. Desta forma, na primeira rodada de interação, quatro especialistas (2, 6, 11 e 22) foram retirados do estudo. Toda a análise foi refeita sem estes especialistas e, quando necessário, novos especialistas foram retirados. Este processo foi finalizado quando não se detectou mais medidas com valores Z_2_ >2 e Z<-2. No total, foram realizadas oito rodadas de interação que resultaram na eliminação de 12 especialistas do estudo.

#### Seleção dos itens

O item foi considerado válido se a correlação de Pearson entre as notas transformadas dos estudantes para um determinado item, com a soma das notas transformadas para todos os itens, foi superior a 0,05^(20)^. A nota transformada dos estudantes foi igual a frequência da nota do estudante na escala *Likert* no grupo de especialistas, dividido pela frequência da moda do grupo de especialistas.

A Tabela 3 ilustra parte das notas dos estudantes utilizando a Escala *Likert,* bem como a moda dos especialistas e respectiva frequência da moda.

**Tabela 3:** Respostas dos estudantes de acordo com a escala *Likert* para cada item do FonoTCS e valor da moda e frequência da moda dos especialistas

	**q1**	**q2**	**...**	**q119**	**q120**
Estudante 1	3	1	...	5	1
Estudante 2	2	3	...	5	4
Estudante 3	2	1	...	5	2
Estudante 4	3	2	...	5	1
Estudante 5	1	2	...	5	2
Estudante 6	3	2	...	5	1
...	...	...	...	...	...
Estudante 30	2	4	...	5	1
Estudante 31	2	4	...	5	1
Estudante 32	3	4	...	5	1
Estudante 33	2	2	...	4	3
Estudante 34	2	1	...	4	2
Estudante 35	1	1	...	2	2
Moda Especialista	2	1	...	5	1
Frequência da Moda	6	9	...	10	6
# Nota Especialista 1	3	9	...	0	6
# Nota Especialista 2	6	2	...	2	3
# Nota Especialista 3	1	0	...	0	0
# Nota Especialista 4	2	1	...	1	3
# Nota Especialista 5	1	1	...	10	1

**Legenda:** q – item do FonoTCS

Na Tabela 4 observa-se as notas transformadas (T) e a respectiva soma das notas transformadas (SNT). Por exemplo, a nota 0,167 para o Estudante 1 para o item 1 é obtida dividindo a frequência da nota 3 (oriunda da Tabela 3) no grupo de especialistas pela frequência da moda no grupo de especialistas, isto é, 1/6. Já a nota transformada 0,222 do estudante 6 para o quesito 2 é obtida pela fração 2/9.

**Tabela 4:** Notas transformadas dos estudantes (NT) e soma das notas transformadas (SNT) para cada item do FonoTCS

	**NTq1**	**NTq2**	**...**	**NTq119**	**NTq120**	**SNT**
Estudante 1	0,167	1,000	...	1,000	1,000	74,630
Estudante 2	1,000	0,000	...	1,000	0,500	67,995
Estudante 3	1,000	1,000	...	1,000	0,500	75,718
Estudante 4	0,167	0,222	...	1,000	1,000	63,192
Estudante 5	0,500	0,222	...	1,000	0,500	86,263
Estudante 6	0,167	0,222	...	1,000	1,000	84,956
...	...	...	...	...	...	...
Estudante 30	1,000	0,111	...	1,000	1,000	90,274
Estudante 31	1,000	0,111	...	1,000	1,000	80,157
Estudante 32	0,167	0,111	...	1,000	1,000	90,977
Estudante 33	1,000	0,222	...	0,100	0,000	65,506
Estudante 34	1,000	1,000	...	0,100	0,500	90,085
Estudante 35	0,500	1,000	...	0,200	0,500	74,453

**Legenda:** q – item do FonoTCS

Após transformar as notas dos estudantes, foram calculadas todas as 120 correlações entre as notas transformadas registradas nas colunas da Tabela 4 (por exemplo, NTq1) com a coluna SNT. Correlações de Pearson maiores que 0,05 indicam que o item deve permanecer no estudo^(20)^.

Nesta etapa, dos 120 itens, 27 itens foram retirados do estudo por apresentarem correlações menores do que 0,05. Todo o processo foi repetido reiteradamente até que todas as correlações fossem maiores do que 0,05. Outros cinco itens foram retirados devido a recomendação de que cada cenário (caso clínico) precisa ter pelo menos dois itens que o caracteriza^(14)^, portanto, após quatro rodadas de interações, 88 itens compuseram o instrumento final.

### Análise dos dados

Após a retirada de especialistas e itens e definição do formato final do FonoTCS, o teste Alfa de Cronbach foi aplicado para medir a consistência interna do teste.

As respostas do FonoTCS foram definidas usando o método de escore agregado^(15)^. A pontuação foi baseada na distribuição das respostas entre os especialistas para cada item. Créditos totais foram dados à resposta modal (mais escolhida pelos especialistas) e créditos parciais foram atribuídos a outras respostas, conforme a proporção de escolha dos fonoaudiólogos especialistas. Respostas não escolhidas pelos juízes receberam pontuação zero.

Posteriormente, os resultados das respostas dos estudantes e dos especialistas foram comparados para avaliar as respostas de ambos os grupos, a partir de análise descritiva dos dados.

Ao finalizar o FonoTCS, o respondente receberá uma nota final. A pontuação total final para o teste é a soma dos pontos obtidos em cada item. Este valor é dividido pelo número de itens do teste e no fim multiplicado por 100 para se obter um escore percentual^(5)^.

## RESULTADOS

Participaram do estudo, após a definição do formato final do FonoTCS, 13 especialistas (52%) dos 25 fonoaudiólogos que responderam o FonoTCS, sendo 12 mulheres (92,0%) e um homem (8,0%) com média de idade de 41,7 anos (DP=6,43). Em relação ao nível de escolaridade a amostra contou com um especialista (08%), oito mestres (61%) e quatro doutores (31%), com tempo médio de atuação clínica generalista de 17,9 anos (DP=6,3).

Dos 35 estudantes do curso de fonoaudiologia, discentes dos 9º e 10º períodos convocados para o Enade, a maioria era do sexo feminino (n=32; 91,4%) com idade média de 25,5 anos (DP=7,76). O tempo médio para a realização do FonoTCS pelos estudantes foi de 58,9 minutos (DP = 8,93).

O instrumento final apresentou 88 itens distribuídos em 28 casos clínicos sendo seis casos para as áreas de conhecimento da fonoaudiologia de audiologia, linguagem, motricidade orofacial/disfagia, e cinco casos para as áreas de voz e saúde coletiva.

As Figuras 1 e 2 ilustram os gráficos de probabilidade normal para os valores Z_2_ e Z dos 13 especialistas, respectivamente. Observa-se que não existe evidência para rejeitar que os dados sejam normais ao nível de significância 5% (p-valor 0,05 para o teste Anderson Darling) e nenhum especialista apresentou valor de Z_2_ >2 e/ou Z< -2.

**Figura 1:** Gráfico de QQ Plot para dados padronizados de Z_2_ dos especialistas que ficaram na versão final (n=13)

**Figura 2:** Gráfico de QQ *Plot* para dados padronizados de Z dos especialistas que ficaram na versão final (n=13)

Utilizando os dados transformados descritos na Tabela 4 com os 88 Itens que permaneceram no estudo, obteve-se um Alfa de Cronbach igual a 0,903 com intervalo de confiança de 95% expresso por 0,86|---|0,95. Estes valores indicam uma alta consistência interna entre os itens do FonoTCS^(21)^.

Podemos observar na Figura 3 que os 13 especialistas apresentaram, como esperado, um desempenho médio maior quando comparado aos estudantes (n=35) além de uma variabilidade menor. Em relação aos estudantes, observou-se uma grande variabilidade e um desempenho médio inferior aos dos especialistas.

**Figura 3:** Avaliação do desempenho dos estudantes e especialistas

Podemos observar que determinados estudantes (19, 20, 31, 32 e 34) apresentaram um desempenho muito bom e comparável ao dos especialistas, podendo ser chamados de “estudantes especialistas” considerando a avaliação dos 88 itens e corroborando um adequado processo de aprendizagem. Por outro lado, alguns estudantes (2, 4, 12, 13, 16, 22, 29 e 33), apresentaram desempenho médio, em termos descritivos, inferior ao dos especialistas.

O formato final do FonoTCS está disponível para acesso gratuito na página da Faculdade de Medicina da Universidade Federal de Minas Gerais, pelo link https://fonotcs.medicina.ufmg.br/.

## DISCUSSÃO

Os resultados desta pesquisa indicaram que é possível avaliar o raciocínio clínico de estudantes e jovens fonoaudiólogos com formação generalista. O FonoTCS foi considerado apropriado para avaliar a capacidade dos examinandos em gerar hipóteses e decidir sobre tomadas de decisão no processo diagnóstico e terapêutico, em contextos de incerteza no campo fonoaudiológico.

O formato inicial do FonoTCS, com conteúdo validado^(12)^, foi composto por 120 itens distribuídos em 30 casos clínicos de seis áreas de conhecimento da Fonoaudiologia (audiologia, linguagem, motricidade orofacial, disfagia, voz e saúde coletiva) com quatro itens cada. Já seu formato final foi composto por 28 casos clínicos com 88 itens, sendo seis casos para as áreas de conhecimento de audiologia, linguagem, motricidade orofacial/disfagia e cinco casos para as áreas de voz e saúde coletiva.

A configuração final do FonoTCS foi definida em duas etapas de análise: 1) seleção dos especialistas a partir dos valores padronizados Z_2_ e Z; e 2) retirada dos itens com correlação item/item total menor que 0,05. Uma característica única dos Testes de Concordância de Scripts (TCSs) é a formação de um painel de especialistas de referência, que tem a função de determinar a pontuação final do teste^(15,17)^.

Os guias para a elaboração dos TCSs indicam que, para se obter testes confiáveis, são necessários de 10 a 15 juízes para alcançar estimativas adequadas de confiabilidade, com um benefício marginal ao se ter mais de 20 especialistas^(13-17,22)^. Dos 25 fonoaudiólogos que responderam ao FonoTCS, 13 especialistas foram mantidos na análise final para a definição das respostas de referência do teste, conforme sugerido pela literatura^(22)^.

Após a análise da correlação item/item total, foram retirados 27 itens (22,5%), e depois cinco por ficarem com cenários (casos clínicos) contendo apenas dois itens, totalizando 32 (26,7%) itens retirados. Segundo a literatura^(14)^, estima-se a retirada de aproximadamente 25% dos itens na elaboração dos TCSs. O uso de três a quatro itens por caso no TCS é justificado por questões teóricas e psicométricas do teste^(23)^. Guias de elaboração de TCSs recomendam de 20 a 25 casos com, no mínimo, 75 itens^(13-16)^, para se alcançar uma aceitável confiabilidade^(14)^. O FonoTCS respeitou todas as recomendações^(13-16)^ para elaboração dos TCSs, apresentando um formato composto por 28 casos clínicos com 88 itens, com três a quatro itens por caso.

O tempo de resposta do teste é de aproximadamente 60 minutos. Pesquisa^(22)^ afirma que TCSs com tempos de teste entre 60 e 90 minutos demonstraram produzir uma confiabilidade de pontuação adequada^(22)^.

O Alfa de Cronbach obtido foi de 0,903, com um intervalo de confiança de 95% variando entre 0,86 e 0,95. Esses valores indicam uma alta consistência interna entre os itens do FonoTCS^(21)^. Considerando que os 88 itens do FonoTCS estão distribuídos em 28 casos clínicos, cada um representando situações distintas e complexas em diferentes áreas da Fonoaudiologia, essa diversidade garante que a alta consistência interna não é resultado de itens homogêneos, minimizando o risco de uma inflação artificial do alfa de Cronbach, e reforçando a validade do instrumento para avaliar o raciocínio clínico do fonoaudiólogo com formação generalista. Outro aspecto a se destacar é que o FonoTCS foi submetido à validação de conteúdo e demonstrou ser um instrumento válido em termos de clareza, pertinência e aspectos éticos^(12)^.

Diferentes TCSs validados para avaliar o raciocínio clínico em medicina, abrangendo situações clínicas de reumatologia^(24)^, otorrinolaringologia^(25)^ e anestesiologia^(26)^, apresentaram valores de Alfa de Cronbach de 0,82^(24,25)^ e 0,79^(26)^, todos dentro da faixa de 0,75 a 0,80 recomendada pela literatura^(14)^.

Na fonoaudiologia, foi desenvolvido um TCS para estudantes falantes do espanhol chileno^(10)^ com 80 itens e uma confiabilidade de 0,67 (mínimo = 0,34; máximo = 0,84). Os autores concluem que, embora o teste tenha apresentado uma baixa confiabilidade global, a estratificação por área de especialidade de cada cenário ofereceu um panorama heterogêneo^(10)^. Destaca-se o fato de os autores^(10)^ não terem seguido todas as normas de elaboração do TCS^(13-16)^, o que talvez, pode ter influenciado nos resultados encontrados.

Os resultados de consistência interna do FonoTCS de 0,903 demonstram que os itens do teste estão correlacionados entre si e, portanto, medem de forma coerente o mesmo constructo, definindo o FonoTCS como um instrumento de avaliação com confiabilidade nas pontuações obtidas^(21)^, e resultados dentro do esperado para os TCSs^(14,15)^.

As respostas fornecidas pelos fonoaudiólogos especialistas definiram a pontuação final do FonoTCS a partir do método de escore agregado^(13-16)^. Para exemplificar este método, suponha que um painel de 15 membros foi solicitado a responder à primeira pergunta do teste, e oito membros selecionaram a resposta +1, cinco membros selecionaram a resposta +2, e dois membros selecionaram a resposta 0. A pontuação para este item seria: resposta +1, 1 ponto (8/8), por ser a resposta modal; resposta +2, 0,625 pontos (5/8); resposta 0, 0,25 pontos (2/8); e respostas -1 e -2 com 0 pontos.

A variabilidade na pontuação das respostas do painel é uma característica inerente ao TCS e tem demonstrado ser um elemento chave na sua capacidade discriminativa e, consequentemente, em sua validade^(16)^.

O método de escore agregado descrito acima é o método mais comumente utilizado nos TCSs^(27)^. No entanto, é importante reconhecer que o método de pontuação ideal para o TCS ainda é debatido^(28)^. O método de escore agregado exige ainda pesquisas psicométricas sobre aspectos como nível de consenso, escala de pontuação e sua relação com a discriminação entre as respostas de profissionais experientes e jovens clínicos^(13)^.

Apesar do debate sobre o melhor método de análise a ser utilizado nos TCSs^(28)^, a variabilidade nas pontuações das respostas é essencial para esses testes. A fundamentação teórica dos TCSs baseia-se no desenvolvimento dos *scripts* a partir da experiência individual do clínico com pacientes, o que resulta em um grau significativo de idiossincrasia, especialmente em casos menos comuns. Portanto, a variabilidade nas respostas, a partir da pontuação do método de escore agregado, é central para a argumentação sobre a validade dos TCSs na capacidade de medir o raciocínio clínico^(15)^.

Como descrito, o método de escore agregado pondera as respostas com base em sua frequência no painel de especialistas, permitindo uma pontuação que reflete a opinião coletiva, com atribuição de pesos proporcionais às respostas menos frequentes.

Um dos principais impactos positivos desse método é sua capacidade de captar a variabilidade nas respostas dos especialistas, o que contribui para a discriminação fina entre diferentes níveis de raciocínio clínico. Esse aspecto é fundamental para a validade do FonoTCS, pois a variabilidade observada reflete as diferentes experiências e formações dos profissionais, especialmente em casos clínicos mais raros ou complexos^(16)^. Essa capacidade discriminativa reforça o argumento de que o FonoTCS é sensível às nuances do raciocínio clínico, uma característica central dos TCSs^(13-16)^.

Por outro lado, um impacto potencialmente negativo do método de escore agregado é que ele pode introduzir alguma subjetividade nas pontuações, principalmente quando há um alto grau de dispersão nas respostas dos especialistas. Essa variação pode dificultar a criação de um padrão claro para todos os casos, o que levanta a necessidade de mais pesquisas psicométricas, como a investigação do nível de consenso necessário para definir respostas corretas e a análise da relação entre esse consenso e a discriminação entre clínicos experientes e jovens profissionais^(13)^.

Portanto, enquanto o método de escore agregado oferece uma maneira robusta de refletir a diversidade de respostas e experiências clínicas no FonoTCS, reconhecemos que ele também apresenta desafios que exigem investigação adicional. Entretanto, os resultados deste estudo indicam que essa abordagem é eficaz em capturar a complexidade do raciocínio clínico fonoaudiológico, contribuindo para a validade do instrumento^(17,28)^.

O FonoTCS será apresentado no formato eletrônico, de acesso livre, para apresentar exames por imagem e para devolutiva dos resultados aos examinandos em tempo real^(14,15)^. Ao finalizar o FonoTCS, o respondente receberá uma nota final calculada da seguinte forma: a pontuação total é obtida somando os pontos de cada item do teste. Esta soma é então dividida pelo número total de itens do teste e multiplicada por 100 para se obter um escore percentual e facilitar a interpretação do resultado^(5)^.

Ao se comparar os resultados dos 13 fonoaudiólogos especialistas com os dos 35 estudantes, os especialistas demonstraram um desempenho superior com uma variabilidade significativamente menor. Alguns estudantes exibiram um excelente desempenho, comparável ao dos especialistas, enquanto outros mostraram um desempenho descritivamente inferior ao dos especialistas. Tais resultados são esperados e demonstram a capacidade dos TCSs discriminar diferentes níveis de raciocínio clínico^(13-16)^.

Como limitação deste estudo, é importante destacar que a elaboração de um TCS requer um investimento significativo de recursos humanos, tempo e dedicação. Em um contexto em que a eficiência é crucial, adaptações rápidas e eficazes dos métodos de avaliação são essenciais para assegurar a qualidade do ensino de profissionais da saúde. Um estudo recente^(29)^ explorou o desenvolvimento de um TCS apoiado por inteligência artificial (IA), ressaltando que o ChatGPT pode ser uma ferramenta promissora na criação desses testes. Esta pesquisa^(29)^ abre novas perspectivas para futuras investigações no campo do TCS.

O cenário atual destaca a necessidade de desenvolver novos TCSs para avaliar o raciocínio clínico nas diversas especialidades da fonoaudiologia. No contexto de formação generalista, o FonoTCS mostrou ser uma ferramenta útil e inovadora para a avaliação do raciocínio clínico de fonoaudiólogos falantes do português brasileiro.

O FonoTCS se mostrou eficaz como ferramenta avaliativa, mas também desempenha um papel importante no processo de ensino-aprendizagem. Ao expor os estudantes a cenários clínicos reais e desafiadores, ele incentiva a aplicação prática do conhecimento teórico, promovendo o desenvolvimento de um raciocínio clínico mais robusto e integrado.

Essa abordagem ativa e contextualizada permite que os alunos reflitam sobre suas decisões e aprimorem suas habilidades de tomada de decisão, aspectos essenciais para sua formação profissional. A relevância do FonoTCS no processo educacional reside em sua capacidade de simular situações de incerteza que são frequentes na prática clínica, contribuindo para uma formação mais completa e alinhada às demandas do mercado de trabalho e à realidade da atuação fonoaudiológica.

## CONCLUSÃO

O FonoTCS é válido e confiável para ser utilizado na avaliação do raciocínio clínico de jovens profissionais e estudantes de fonoaudiologia com formação generalista, falantes do português brasileiro.
